# Supplementary material for: Oxidative stress causes a reversible decrease of deubiquitylases activity in old vertebrate brains
Source: Nat Commun. 2026 Apr 21;17:3653. doi: 10.1038/s41467-026-71921-y (PMC13100221; doi:10.1038/s41467-026-71921-y)

Oxidative stress causes a reversible decrease of deubiquitylases activity in old vertebrate brains

Amit Kumar Sahu[1,2], Alberto Minetti[1,3], Domenico Di Fraia[1,2], Antonio Marino[1,4], Patrick Rainer Winterhalter[5], Daniela Giustarini[6], Ranieri Rossi[6], Andreas Simm[5], Francesco Neri[1,7], Federico Galvagni[6], Christoph Gerhardt[8], Thorsten Pfirrmann[8,*], Alessandro Ori[1,9,*]

[1] Leibniz Institute on Aging - Fritz Lipmann Institute (FLI), Jena, Germany
[2] Current address: Cologne Excellence Cluster for Cellular Stress Response in Aging-Associated Diseases (CECAD), University of Cologne, Cologne, Germany
[3] Current address: Department of Neurosurgery, University Hospital Erlangen, Friedrich-Alexander University Erlangen Nuremberg, Erlangen, Germany
[4] Current address: Proteomics Research Infrastructure, University of Copenhagen, Copenhagen, Denmark
[5] Clinic for Heart Surgery (UMH), Martin-Luther-University Halle-Wittenberg, Halle (Saale), Germany
[6] Department of Biotechnology, Chemistry and Pharmacy, University of Siena, Siena, Italy
[7] Department of Life Sciences and Systems Biology, University of Turin, Torino, Italy
[8] Institute for Molecular Medicine, Department of Medicine, Health and Medical University Potsdam, 14471 Potsdam, Germany
[9] Current address: Genentech Inc., South San Francisco, CA 94080, USA
[*] Correspondence should be addressed to: thorsten.pfirrmann@hmu-potsdam.de , alessandro.ori@leibniz-fli.de

Supplementary Information

The PDF file includes:
Supplementary Figures S1 to S6
Supplementary Table 1
Uncropped Western blot images

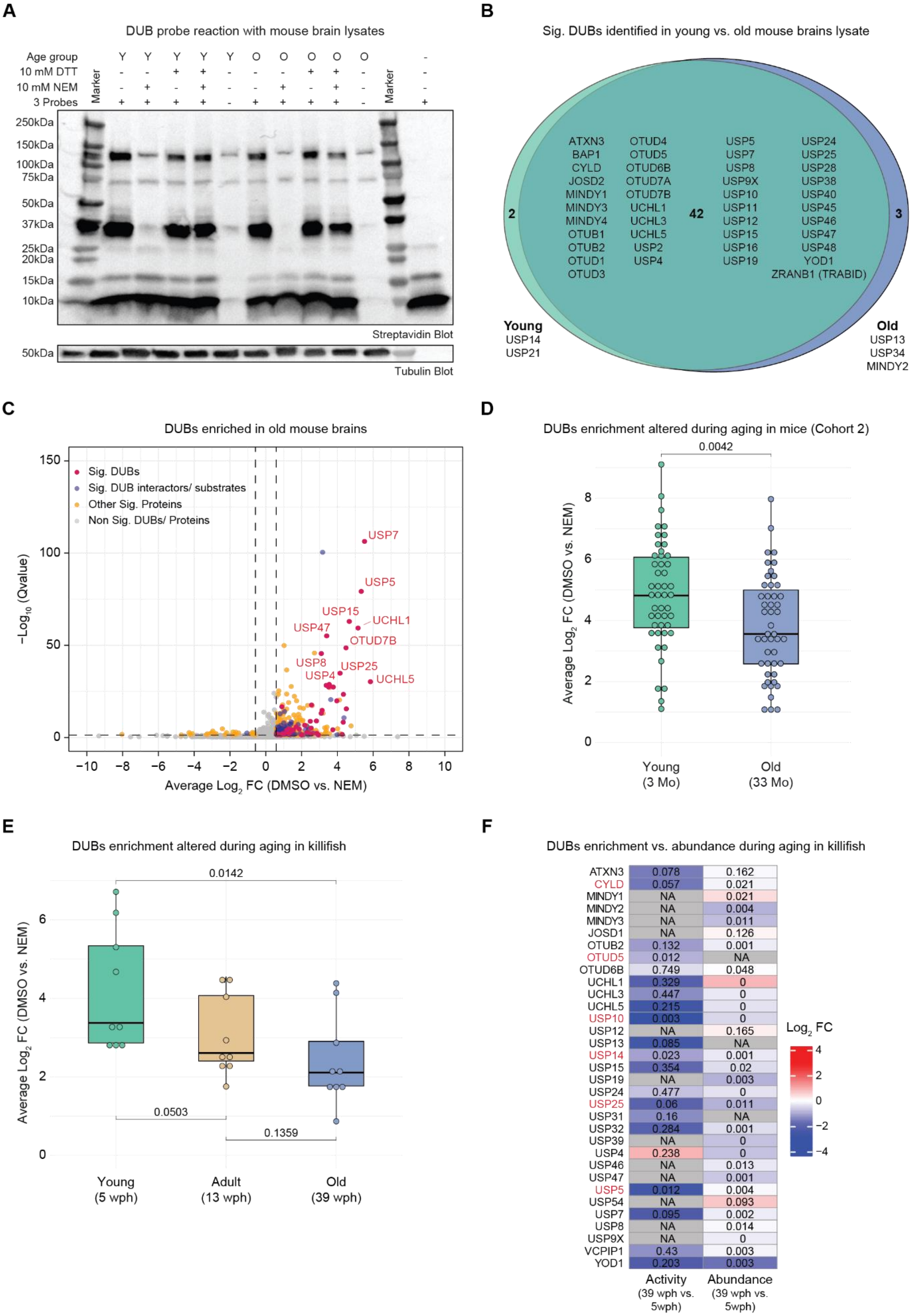
A
DUB probe reaction with mouse brain lysates
Age group
10 mM DTT
10 mM NEM
3 Probes
Marker
Y Y Y Y Y O O O O O -
250kDa
150kDa
100kDa
75kDa
50kDa
37kDa
25kDa
20kDa
15kDa
10kDa
Streptavidin Blot
50kDa
Tubulin Blot
B
Sig. DUBs identified in young vs. old mouse brains lysate
ATXN3
BAP1
CYLD
JOSD2
MINDY1
MINDY3
MINDY4
OTUB1
OTUB2
OTUD1
OTUD3
OTUD4
OTUD5
OTUD6B
OTUD7A
OTUD7B
UCHL1
UCHL3
UCHL5
USP2
USP4
USP5
USP7
USP8
USP9X
USP10
USP11
USP12
USP15
USP16
USP19
USP24
USP25
USP28
USP38
USP40
USP45
USP46
USP47
USP48
YOD1
ZRANB1 (TRABID)
2
42
3
Young
USP14
USP21
Old
USP13
USP34
MINDY2
C
DUBs enriched in old mouse brains
Sig. DUBs
Sig. DUB interactors/ substrates
Other Sig. Proteins
Non Sig. DUBs/ Proteins
USP7
USP5
USP15
UCHL1
USP47
OTUD7B
USP8
USP25
USP4
UCHL5
−Log10 (Qvalue)
Average Log2 FC (DMSO vs. NEM)
D
DUBs enrichment altered during aging in mice (Cohort 2)
0.0042
Average Log2 FC (DMSO vs. NEM)
Young
(3 Mo)
Old
(33 Mo)
E
DUBs enrichment altered during aging in killifish
0.0142
0.0503
0.1359
Average Log2 FC (DMSO vs. NEM)
Young
(5 wph)
Adult
(13 wph)
Old
(39 wph)
F
DUBs enrichment vs. abundance during aging in killifish
ATXN3 0.078 0.162
CYLD 0.057 0.021
MINDY1 NA 0.021
MINDY2 NA 0.004
MINDY3 NA 0.011
JOSD1 NA 0.126
OTUB2 0.132 0.001
OTUD5 0.012 NA
OTUD6B 0.749 0.048
UCHL1 0.329 0
UCHL3 0.447 0
UCHL5 0.215 0
USP10 0.003 0
USP12 NA 0.165
USP13 0.085 NA
USP14 0.023 0.001
USP15 0.354 0.02
USP19 NA 0.003
USP24 0.477 0
USP25 0.06 0.011
USP31 0.16 NA
USP32 0.284 0.001
USP39 NA 0
USP4 0.238 0
USP46 NA 0.013
USP47 NA 0.001
USP5 0.012 0.004
USP54 NA 0.093
USP7 0.095 0.002
USP8 NA 0.014
USP9X NA 0
VCPIP1 0.43 0.003
YOD1 0.203 0.003
Activity
(39 wph vs.
5wph)
Abundance
(39 wph vs.
5wph)
Log2 FC
4
2
0
−2
−4

Supplementary Figure 1: DUB activity in mouse and killifish brains during aging.
(A) Immunoblot validation of DUB probe labeling in young and old mouse brain lysates pre-treated with DMSO, NEM, or DTT. Immunoblots were repeated for all biological replicates used in DUB activity profiling (N = 3). (B) Venn diagram showing overlap of significantly enriched active DUBs in young and old mouse brain lysates normalized to NEM-treated controls (cohort 1; |average $\log_2$ FC| > 0.58; Qvalue < 0.05; N = 3). (C) Volcano plot of proteins enriched from old mouse brains (cohort 1, N = 3). Vivid pink dots indicate active DUBs; deep indigo dots denote known DUB substrates and interactors [18,19]; vivid yellow dots indicate other significantly co-enriched proteins; light grey dots represent non-significant proteins. Seven non-DUB proteins were excluded for clarity. Differential abundance was assessed using Spectronaut (Qvalue). Dashed lines indicate thresholds (|average $\log_2$ FC| > 0.58; Qvalue < 0.05). (D) Boxplot showing DUB enrichment in young (3 months) and old (33 months) mouse brains (cohort 2). 47 DUBs detected in both groups are shown (N = 3; |average $\log_2$ FC| > 0.58; Qvalue < 0.05; two-tailed Wilcoxon rank-sum test). (E) Boxplot showing DUB enrichment in young (5 wph), adult (13 wph), and old (39 wph) killifish brains. 9 DUBs detected in all age groups are shown (N = 3; |average $\log_2$ FC|) > 0.58; Qvalue < 0.05; two-tailed Wilcoxon rank-sum test; wph = weeks post hatching). In both box plots, data are shown as the median (central line) and the interquartile range (25th–75th percentiles, box limits). Whiskers extend to 1.5× the interquartile range, and individual measurements of DUB enrichment are overlaid as dots. (F) Heatmap comparing age-associated changes in DUB activity (this study; 39 vs. 5 wph; N = 3) and protein abundance (DIA proteomics from [29]) in killifish brains. Values represent Pvalues from two-tailed unpaired t-tests with Welch's correction. 'NA' indicates DUBs not detected. DUBs highlighted in red exhibit significant (Pvalue < 0.05) age-associated activity changes. N refers to the number of biological replicates used in the experiment. Related to Supplementary Data 1. Source data are provided as a Source Data file.

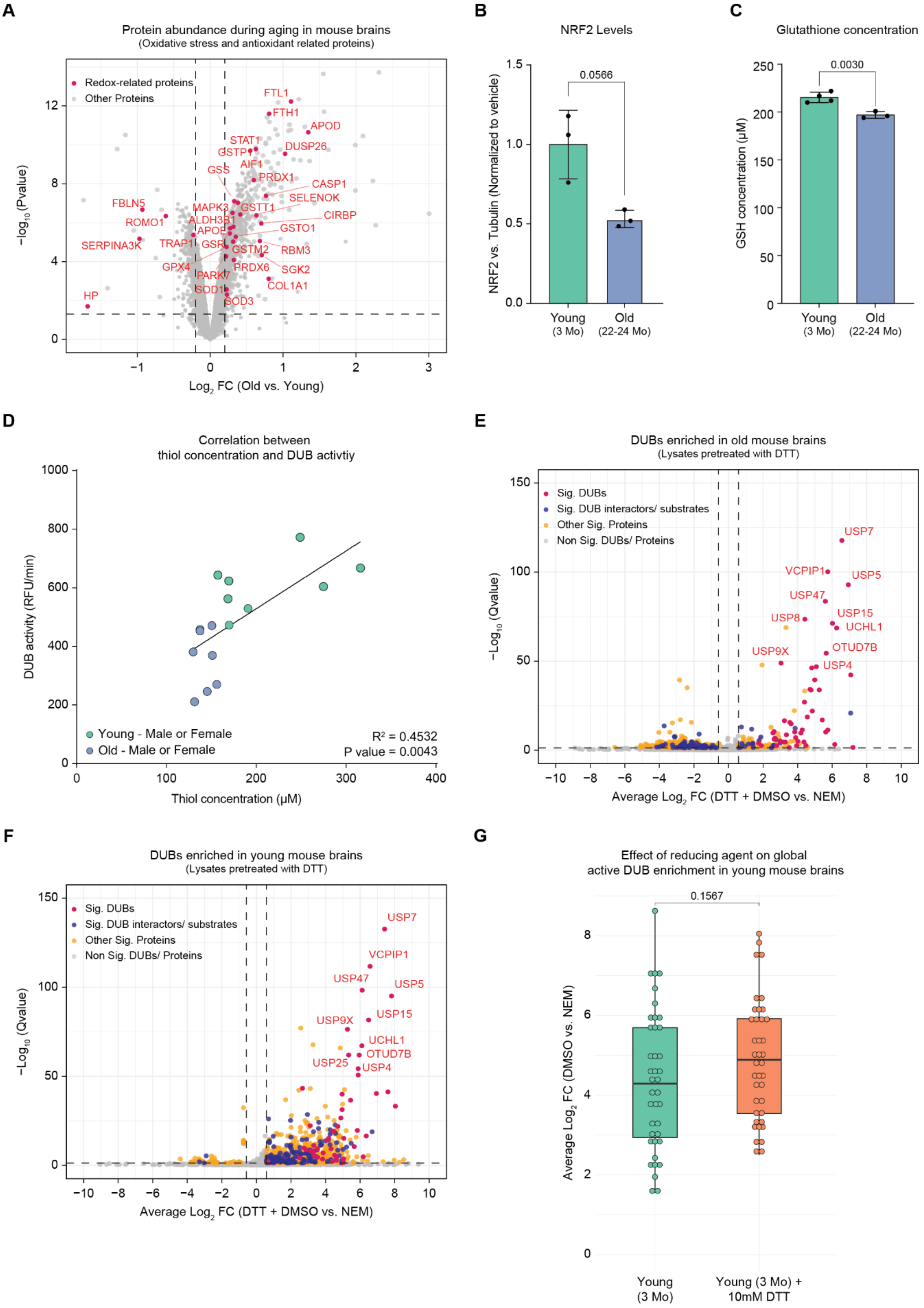
A
Protein abundance during aging in mouse brains
(Oxidative stress and antioxidant related proteins)
Redox-related proteins
Other Proteins
FTL1
FTH1
APOD
STAT1
GSTP1
DUSP26
AIF1
GSS
PRDX1
CASP1
SELENOK
FBLN5
MAPK3
GSTT1
CIRBP
ROMO1
ALDH3B1
APOE
GSTO1
SERPINA3K
TRAP1
GSR
GSTM2
RBM3
GPX4
PRDX6
SGK2
PARK7
COL1A1
SOD1
SOD3
HP
−log10 (Pvalue)
Log2 FC (Old vs. Young)
B
NRF2 Levels
0.0566
NRF2 vs. Tubulin (Normalized to vehicle)
Young (3 Mo)
Old (22-24 Mo)
C
Glutathione concentration
0.0030
GSH concentration (µM)
Young (3 Mo)
Old (22-24 Mo)
D
Correlation between thiol concentration and DUB activtiy
DUB activity (RFU/min)
Young - Male or Female
Old - Male or Female
R² = 0.4532
P value = 0.0043
Thiol concentration (µM)
E
DUBs enriched in old mouse brains
(Lysates pretreated with DTT)
Sig. DUBs
Sig. DUB interactors/ substrates
Other Sig. Proteins
Non Sig. DUBs/ Proteins
USP7
VCPIP1
USP5
USP47
USP8
USP15
UCHL1
OTUD7B
USP9X
USP4
−Log10 (Qvalue)
Average Log2 FC (DTT + DMSO vs. NEM)
F
DUBs enriched in young mouse brains
(Lysates pretreated with DTT)
Sig. DUBs
Sig. DUB interactors/ substrates
Other Sig. Proteins
Non Sig. DUBs/ Proteins
USP7
VCPIP1
USP47
USP5
USP9X
USP15
UCHL1
OTUD7B
USP25
USP4
−Log10 (Qvalue)
Average Log2 FC (DTT + DMSO vs. NEM)
G
Effect of reducing agent on global active DUB enrichment in young mouse brains
0.1567
Average Log2 FC (DMSO vs. NEM)
Young (3 Mo)
Young (3 Mo) + 10mM DTT

Supplementary Figure 2: Oxidative stress-mediated reversible cysteine oxidation modulates DUB activity during brain aging.

(A) Volcano plot of redox-related protein abundance changes in old (33 months) vs. young (3 months) C57BL/6J male mouse brains (N = 5; proteomics data from [5]). Red dots indicate redox-related proteins. Dashed lines indicate thresholds (|average $\log_2$ FC| > 0.2; Pvalue < 0.05). (B) NRF2 protein levels (see Fig. S6A for the blot) in brain lysates from young (3 months, N = 3) and old (22–24 months, N = 3) C57BL/6J female mice. Two-tailed unpaired t-test with Welch's correction. Data are shown as mean ± SD. (C) GSH concentrations in brain lysates from young (3 months, N = 4) and old (22–24 months, N = 3) C57BL/6J female mice. Two-tailed unpaired t-test with Welch's correction. Data are shown as mean ± SD. (D) Linear regression analysis between thiol concentration (μM, x-axis) and DUB activity (RFU/min, y-axis) from both male and female mice. The solid line represents the best-fit regression line (R^2 = 0.45; Pvalue = 0.0043). Data used from figures 1A and 2A. (E) Volcano plot of proteins enriched from old mouse brain lysates treated with 10 mM DTT (N =3). Seven non-DUB proteins, one DUB interactor, and three non-significant proteins were excluded for clarity. (F) Volcano plot of proteins enriched from young mouse brain lysates treated with 10 mM DTT (N = 3). Three non-DUB proteins and three non-significant proteins were excluded for clarity. Vivid pink dots indicate active DUBs; deep indigo dots mark known DUB substrates and interactors [18,19]; vivid yellow dots denote other significantly co-enriched proteins; light grey dots represent non-significant proteins. Differential abundance was assessed using Spectronaut (Qvalue). Dashed lines indicate thresholds (|average $\log_2$ FC| > 0.58; Qvalue < 0.05). (G) Boxplot of individual active DUB enrichment in young mouse brains with or without 10 mM DTT treatment. 39 DUBs detected under both conditions are shown (N = 3; |average $\log_2$ FC| > 0.58; Qvalue < 0.05; two-tailed Wilcoxon rank-sum test). Data are shown as the median (central line) and the interquartile range (25th–75th percentiles, box limits). Whiskers extend to 1.5× the interquartile range, and individual measurements of DUB enrichment are overlaid as dots. N refers to the number of biological replicates used in the experiment. Related to Supplementary Data 1. Source data are provided as a Source Data file.

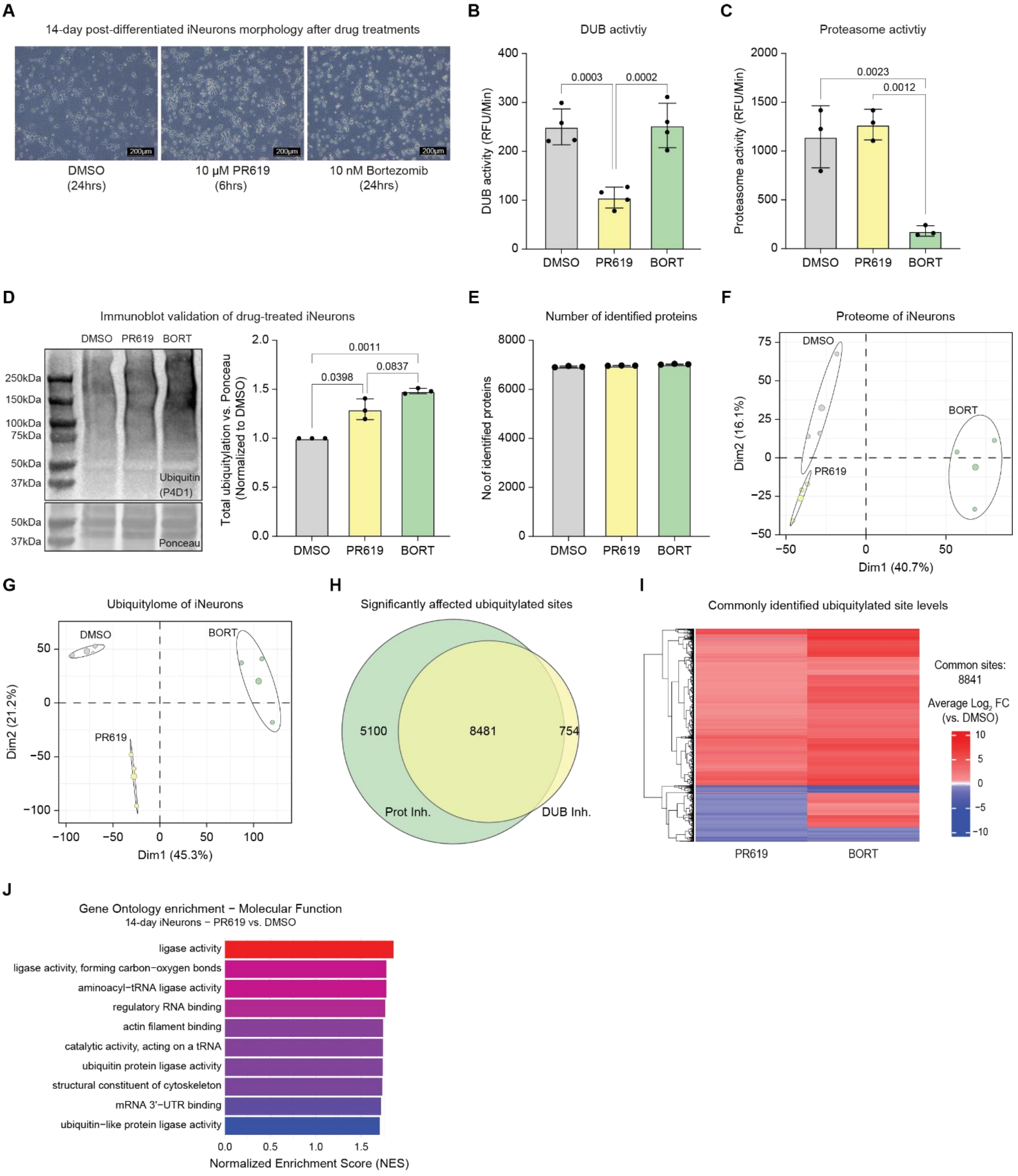

Supplementary Figure 3: Impact of DUB inhibition on proteome and ubiquitylome in iNeurons. (A) Representative images showing the morphology of 14 days post-differentiated iNeurons after treatment with DMSO, 10 μM PR619, or 10 nM Bortezomib for the indicated durations (magnification = 10X; scale = 200 μm; repeated across N = 3). (B) DUB activity in iNeurons (N = 4). (C) Proteasome activity in iNeurons (N = 3) following drug treatment. In both panels, one-way ANOVA was used, RFU = Relative Fluorescence Units. Data are shown as mean ± SD. (D) Left: Immunoblot analysis showing increased levels of total ubiquitylated proteins in iNeurons following PR619 and Bortezomib treatments. Total protein levels were assessed using Ponceau staining. Immunoblots were repeated for all biological replicates used in ubiquitylated site enrichment with K-ε-GG antibody and proteome analysis

(N = 3). Right: Quantification of total ubiquitylation normalized to total protein levels across treatment conditions (N = 3; two-tailed unpaired t-test with Welch's correction). Data are shown as mean ± SD. (E) Number of identified proteins in iNeurons treated with the indicated drugs (N = 3). Data are shown as mean ± SD. (F) PCA of proteome changes in iNeurons (N = 3). (G) PCA of ubiquitylome changes in iNeurons (N = 3). Ellipses represent 95% confidence intervals for highlighted drug treatments in both plots. Percent variance explained by each principal component is indicated (N = 3). (H) Venn diagram showing the overlap in the number of differentially enriched ubiquitylated sites between DUB inhibition (PR619 vs. DMSO) and proteasome inhibition (Bortezomib vs. DMSO) (N = 3; |average $\log_2$ FC| > 0.58; Qvalue < 0.05). (I) Heatmap displaying hierarchical clustering (based on Euclidean distance) of 8,481 ubiquitylated sites commonly identified in DUB- and proteasome-inhibited iNeurons. The column represents treatment conditions, and rows show average $\log_2$ FC intensity of each ubiquitylated site compared to DMSO (N = 3; |average $\log_2$ FC| > 0.58; Qvalue < 0.05). (J) Gene Set Enrichment Analysis (GSEA) of ubiquitylated sites altered in DUB-inhibited iNeurons (PR619 vs. DMSO). The top 10 Gene Ontology (GO) terms associated with increased ubiquitylation, ranked by normalized enrichment score (NES), are shown. N refers to the number of biological replicates used in the experiment. Related to Supplementary Data 2. Source data are provided as a Source Data file.

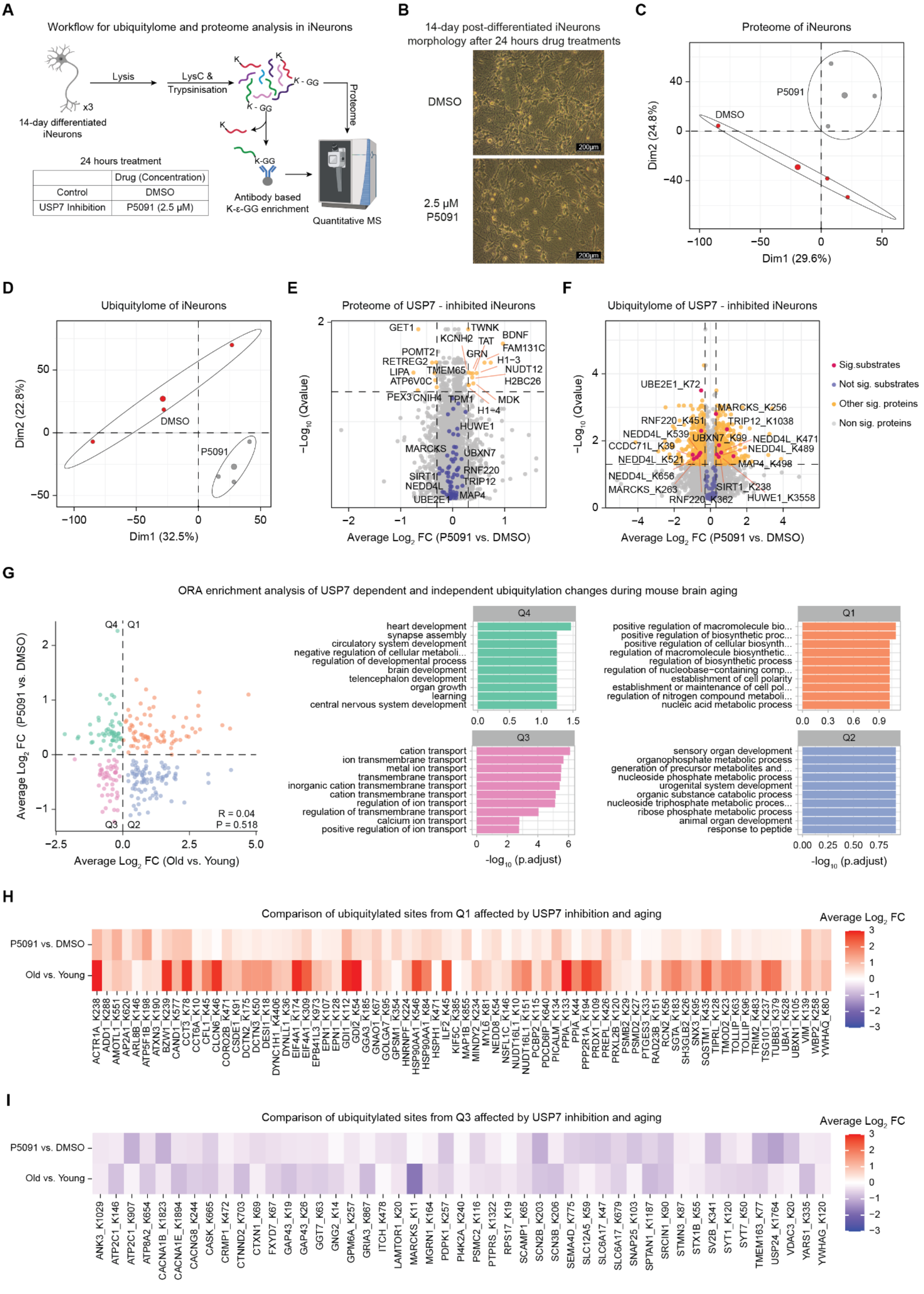

Supplementary Figure 4: Impact of USP7 inhibition on ubiquitylome of iNeurons

(A) Schematic illustrating the K-ε-GG antibody-mediated ubiquitylated peptide enrichment and total proteome analysis of iPSC-derived iNeurons treated either with DMSO or 2.5 μM P5091 (USP7 inhibitor) for 24 hours (N = 3). (B) Representative images showing the morphology of 14 days post-differentiated iNeurons after treatment with DMSO or 2.5 μM P5091 (USP7 inhibitor) for 24 hours (magnification = 20X; scale = 200 μm; repeated across N = 3). (C) PCA of proteome changes in iNeurons (N = 3). (D) PCA of ubiquitylome changes in iNeurons (N = 3). Ellipses represent 95% confidence intervals for highlighted drug treatments in both plots. Percent variance explained by each principal component is indicated (N = 3). (E) Volcano plot of protein abundance changes in USP7-inhibited neurons (N = 3). (F) Volcano plot of ubiquitylated peptide changes in USP7-inhibited neurons (N = 3). In both volcano plots, vivid pink dots indicate significantly altered substrates of USP7 from [19]; deep indigo dots mark substrates of USP7 that are not significantly altered; vivid yellow dots denote other significantly altered proteins or peptides; light grey dots indicate non-significant proteins or peptides. Differential abundance was assessed using Spectronaut (Qvalue). Dashed lines indicate thresholds (|average $\log_2$ FC| > 0.3; Qvalue < 0.05). (G) Left: Scatter plot comparing differentially enriched ubiquitylated sites between mouse aging (old vs. young; x-axis; from [5]) and USP7-inhibited iNeurons (P5091 vs. DMSO; y-axis; this study; N = 3). Right: Quadrant-based Over Representation Analysis (ORA) of the top 10 biological processes. Data includes ubiquitylated site changes with adj.pvals < 0.05 (for mouse) and Qvalue < 0.05 (for iNeurons). (H) Heatmap representing the comparison of average $\log_2$ FC of all enriched ubiquitylated sites from Quadrant 1 (Q1) in (G) between mouse aging and USP7-inhibited iNeurons. (I) Heatmap representing the comparison of average $\log_2$ FC of all enriched ubiquitylated sites from Quadrant 3 (Q3) in (G) between mouse aging and USP7-inhibited iNeurons. N refers to the number of biological replicates used in the experiment. For both heatmaps, only differentially enriched sites with adj.pvals < 0.05 (for mouse) and Qvalue < 0.05 (for iNeurons) were used. Related to Supplementary Data 2. Created in BioRender. Sahu, A. (2026) https://BioRender.com/5q3eb5x.

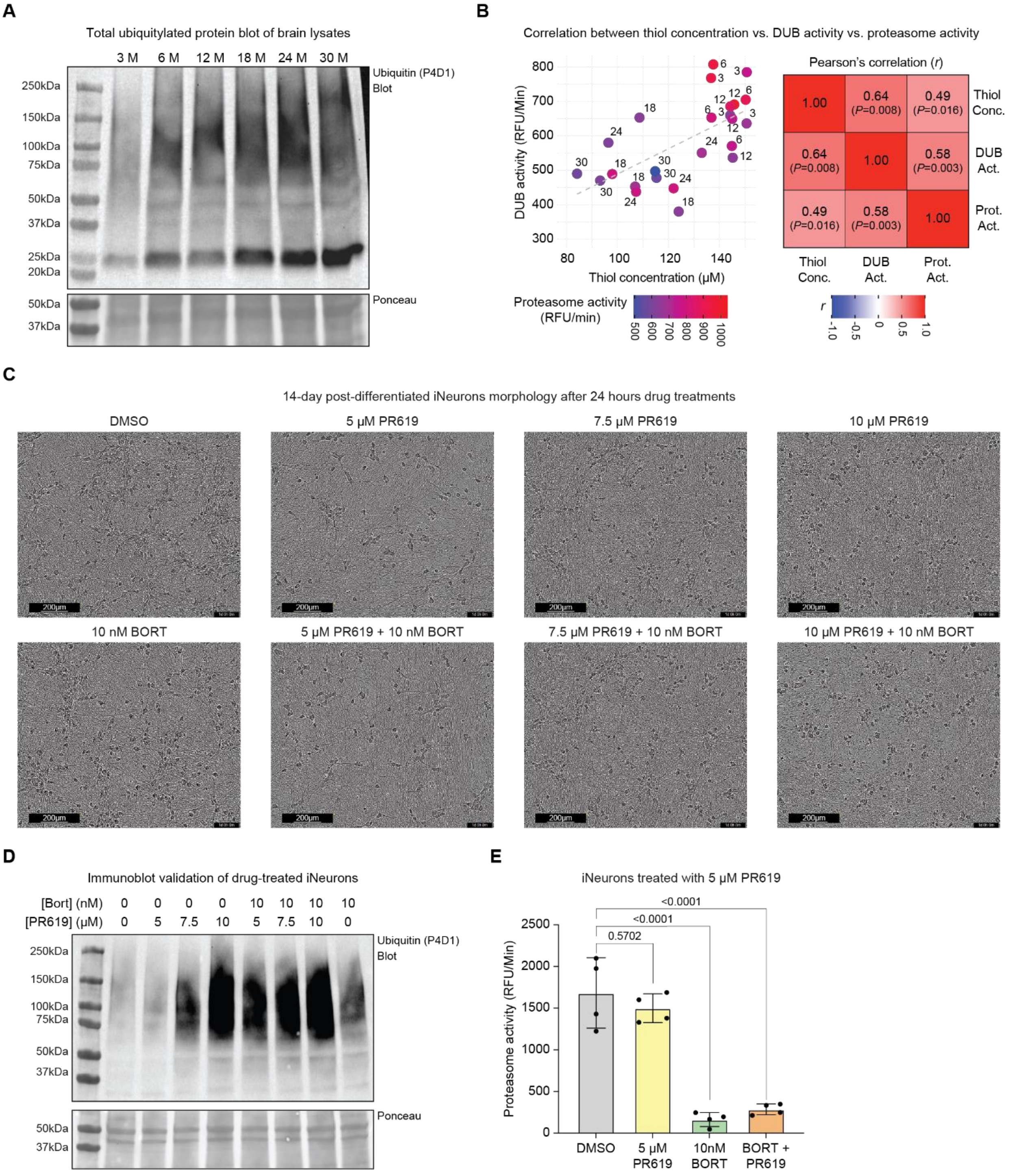

Supplementary Figure 5: Effects of DUB inhibition on ubiquitylated protein levels and proteasome activity in mouse brain and iNeurons.

(A) Immunoblot analysis of total ubiquitylated protein levels in mouse brains across the indicated age groups. Total protein levels were assessed using Ponceau staining (N = 3 for females; N = 4 for males). (B) Left: Scatter plot showing the relationship between thiol concentration (X-axis), DUB activity (Y-axis), and proteasome activity (represented by bubble color) in male mouse brains of the indicated age groups. Right: Pearson's correlation analysis of the molecular changes associated with brain aging. Data used from figures 4B, 4C, and 4E. (C) Representative images showing the morphology of 14 days post-differentiated iNeurons after 24 hours treatment with varying concentrations of PR619, with or without 10 nM Bortezomib (magnification = 10X; scale = 200 μm; repeated across N = 3). (D)

Immunoblot analysis validating changes in total ubiquitylated protein levels in iNeurons following treatment with increasing concentrations of PR619 and 10 nM bortezomib. Total protein levels were assessed using Ponceau staining. (E) Proteasome activity of iNeurons treated for 24 hours with 5 µM PR619, 10 nM Bortezomib, or their combination (N = 4; one-way ANOVA; RFU = Relative Fluorescence Units). Data are shown as mean ± SD. N refers to the number of biological replicates used in the experiment. Source data are provided as a Source Data file.

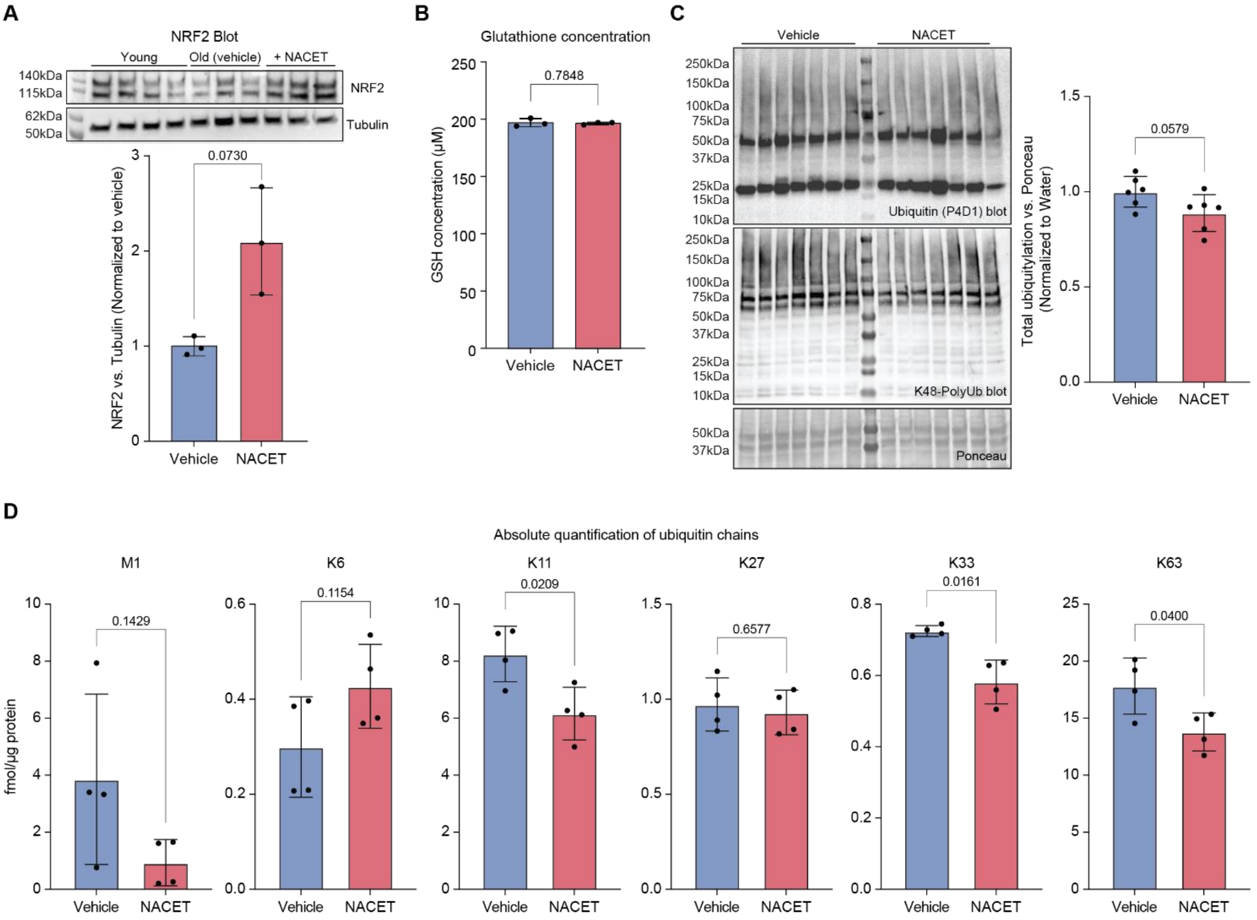

Supplementary Figure 6: NACET treatment rescues brain ubiquitylation in aged mice

(A) Upper: Immunoblot of NRF2 protein levels in young (3 months), old (22-24 months), and old C57BL/6J female mice treated with NACET. Lower: NRF2 protein abundance quantification (lower band) in NACET vs. vehicle-treated aged mouse brains. Total protein levels were assessed using Tubulin (N = 3; two-tailed unpaired t-test with Welch's correction). (B) Glutathione (GSH) concentrations in NACET vs. vehicle-treated aged mouse brains (N = 3; two-tailed unpaired t-test with Welch's correction). (C) Left: Immunoblot of total and K48 polyubiquitylated protein levels. Total protein levels were assessed using Ponceau staining. Right: Total ubiquitylated protein abundance quantification normalized to total protein levels. Replicate 1 from the vehicle and replicate 4 from the NACET-treated animals were removed from both quantification analyses (N = 6). (D) AQUA-PRM of ubiquitin chain linkages in NACET vs. vehicle-treated aged mouse brains (N = 4; two-tailed unpaired t-test with Welch's correction). Data are shown as mean ± SD in all panels. N refers to the number of biological replicates used in the experiment. Related to Supplementary Data 3. Source data are provided as a Source Data file.

Dataset	Spectronaut version	Library size
DUB activity profiling - Killifish	18.3.23	1,540 proteins
DUB activity profiling - Mouse (Cohort 1)	18.6.23	2,549 proteins
DUB activity profiling - Mouse (Cohort 2)	18.4.23	664 proteins
Ubiquitylome - iNeurons (PR619-treated)	18.7.24	27,572 peptides (5,410 proteins)
Proteome - iNeurons (PR619-treated)	18.7.24	6,906 proteins
Ubiquitylome - iNeurons (P5091-treated)	19.9.25	25,076 modified peptides (4,980 proteins)
Proteome - iNeurons (P5091-treated)	19.9.25	6,757 proteins

Supplementary Table 1: MS data used for spectral library generation and DIA analysis in Spectronaut. Library size refers to average profile protein group numbers for proteome datasets, and to quantified ubiquitin-modified peptides and corresponding protein groups for ubiquitylome datasets.

Supplementary Figure 1A: DUB probe reaction with mouse brain lysates

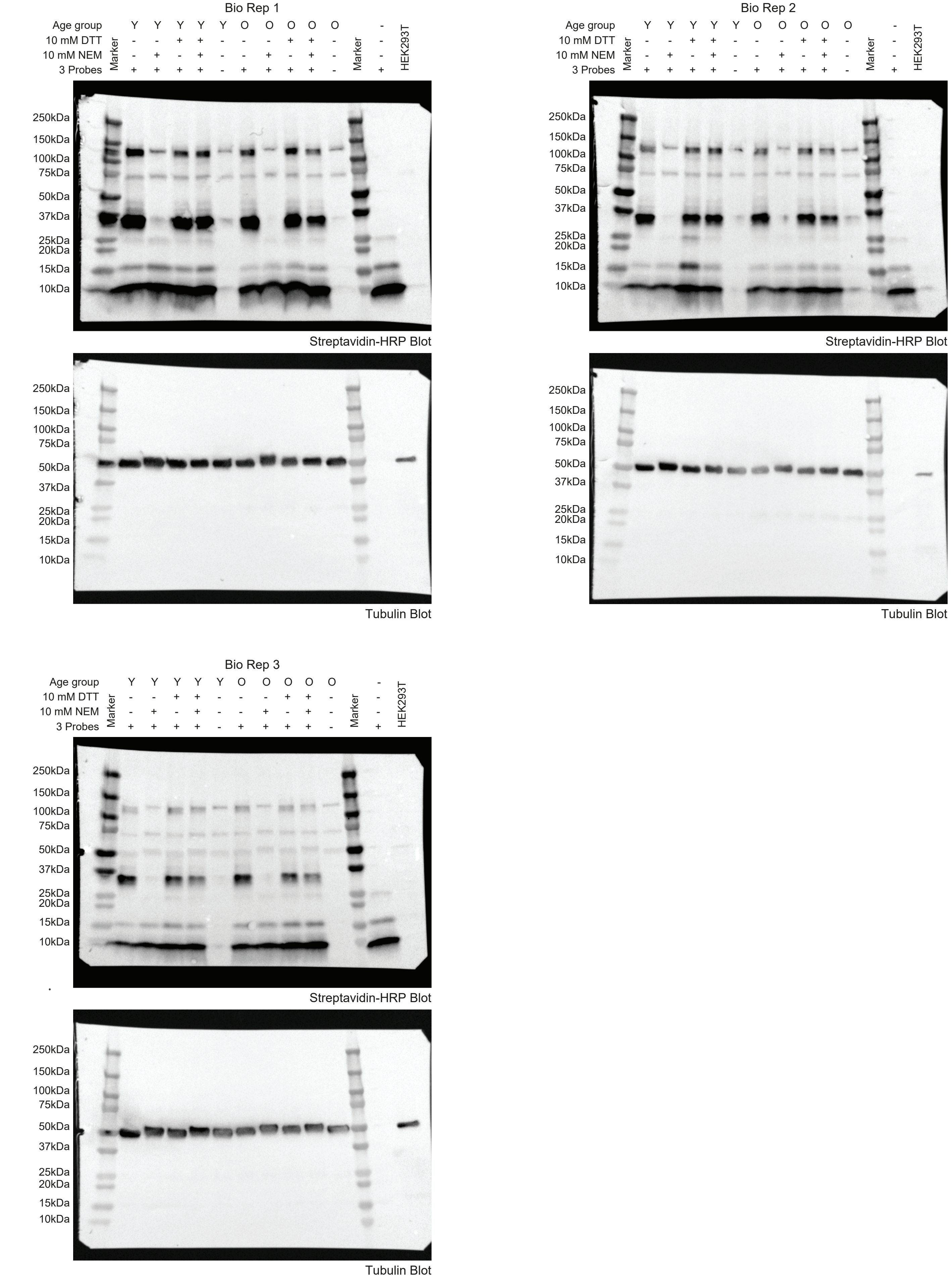

Supplementary Figure 3D: Immunoblot validation of drug-treated iNeurons

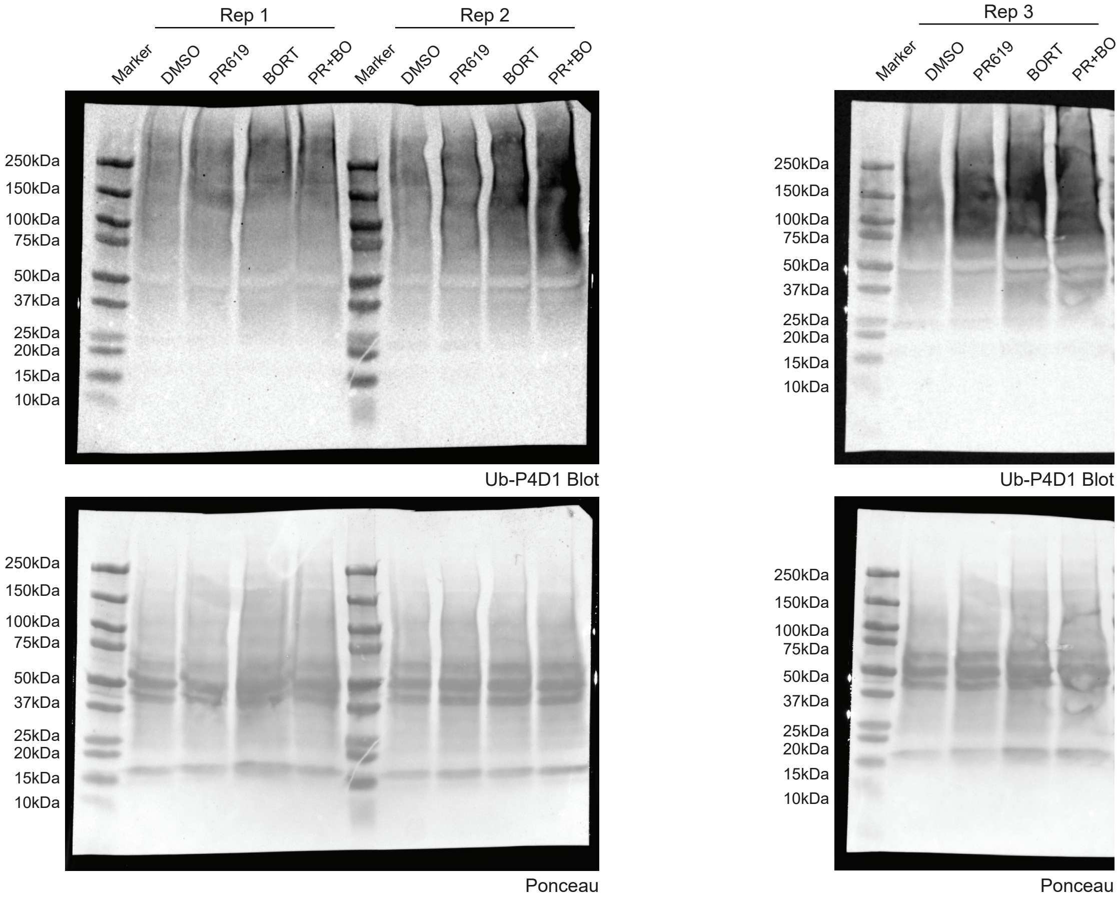

Treatments:
DMSO - 24 hours
PR619 - 6 hours
Bortezomib (BORT) - 24 hours

PR+BO (PR619 + Bortezomib) -
PR619 was added after 18 hours
of Bortezomib treatment (as a control)

Supplementary Figure 5A: Total ubiquitylated protein blot of brain lysates

Male

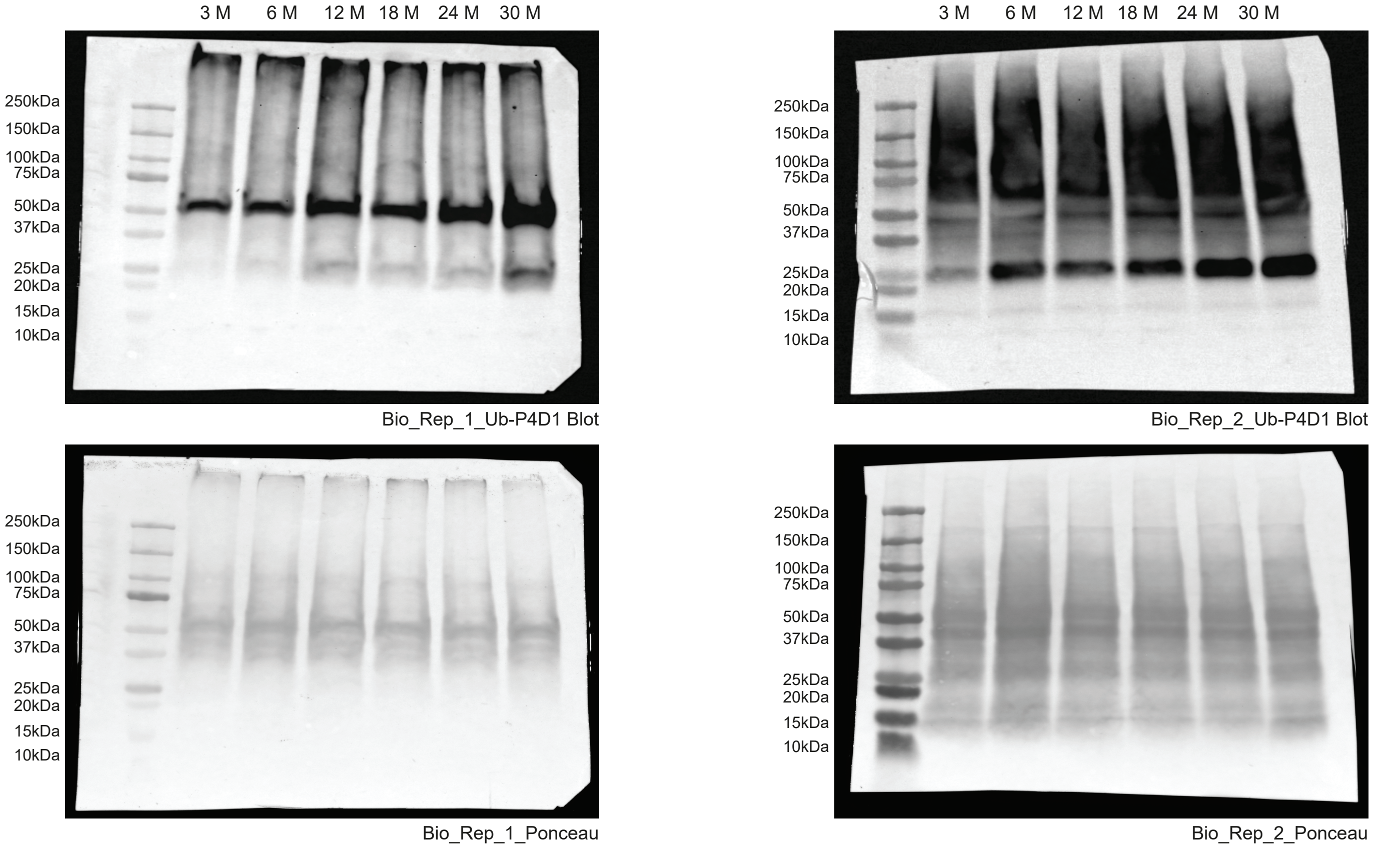

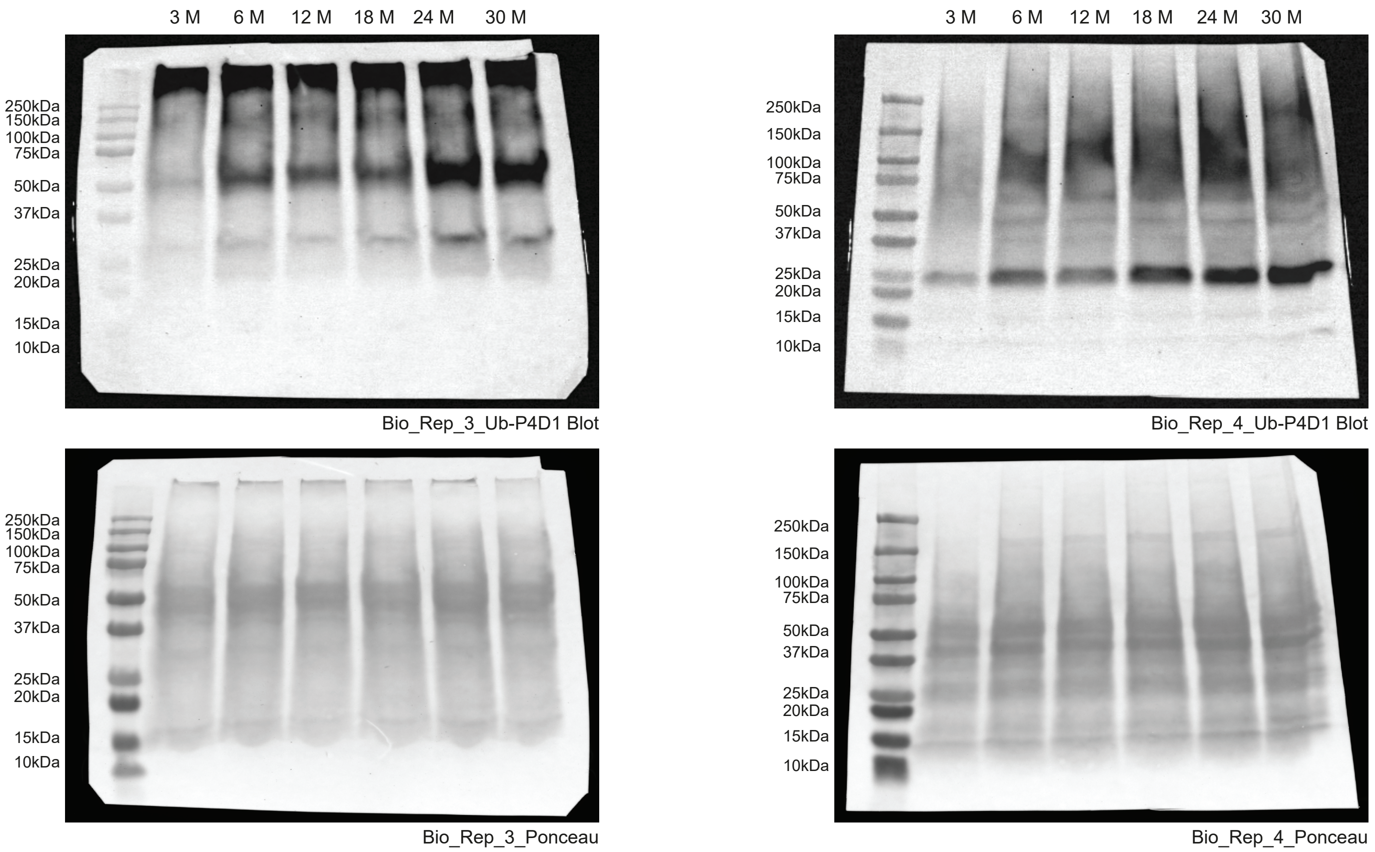
3 M
6 M
12 M
18 M
24 M
30 M
250kDa
150kDa
100kDa
75kDa
50kDa
37kDa
25kDa
20kDa
15kDa
10kDa
Bio_Rep_3_Ub-P4D1 Blot
Bio_Rep_4_Ub-P4D1 Blot
Bio_Rep_3_Ponceau
Bio_Rep_4_Ponceau

Female

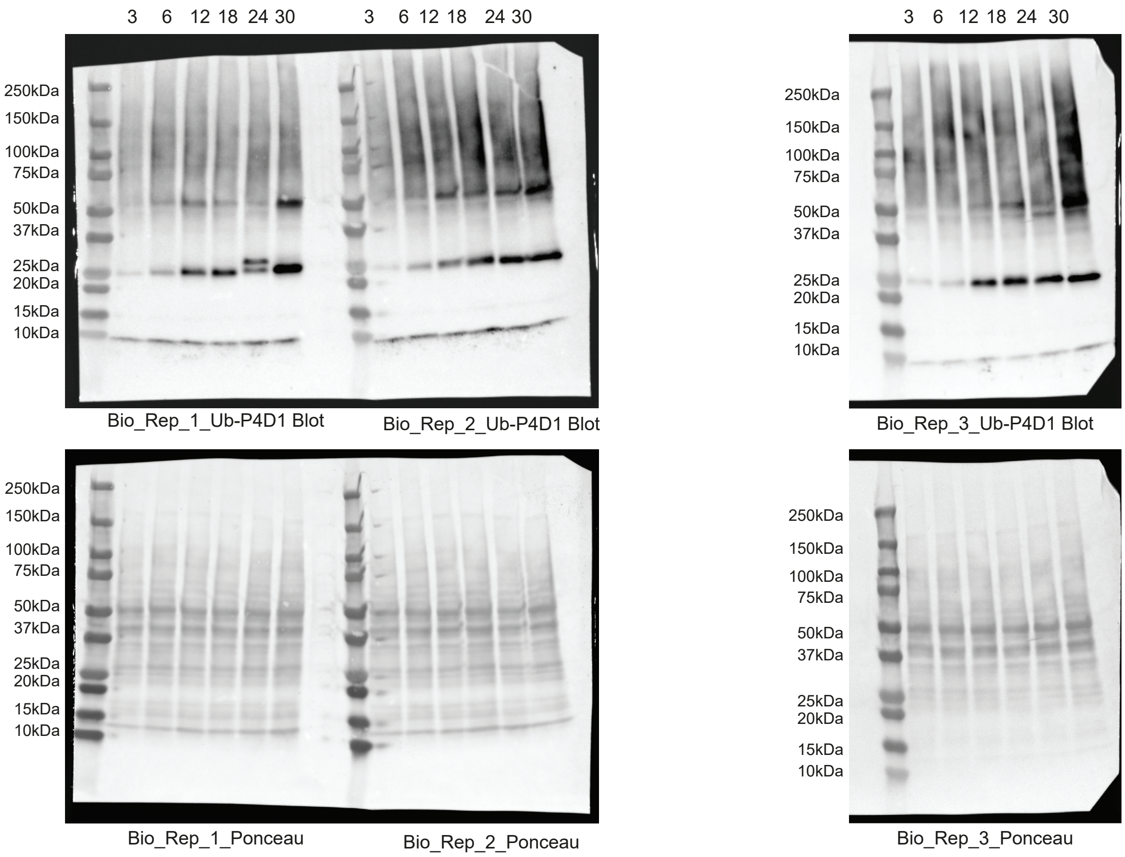
3
6
12
18
24
30
250kDa
150kDa
100kDa
75kDa
50kDa
37kDa
25kDa
20kDa
15kDa
10kDa
Bio_Rep_1_Ub-P4D1 Blot
Bio_Rep_2_Ub-P4D1 Blot
Bio_Rep_3_Ub-P4D1 Blot
Bio_Rep_1_Ponceau
Bio_Rep_2_Ponceau
Bio_Rep_3_Ponceau

Supplementary Figure 5D: Immunoblot validation of drug-treated iNeurons

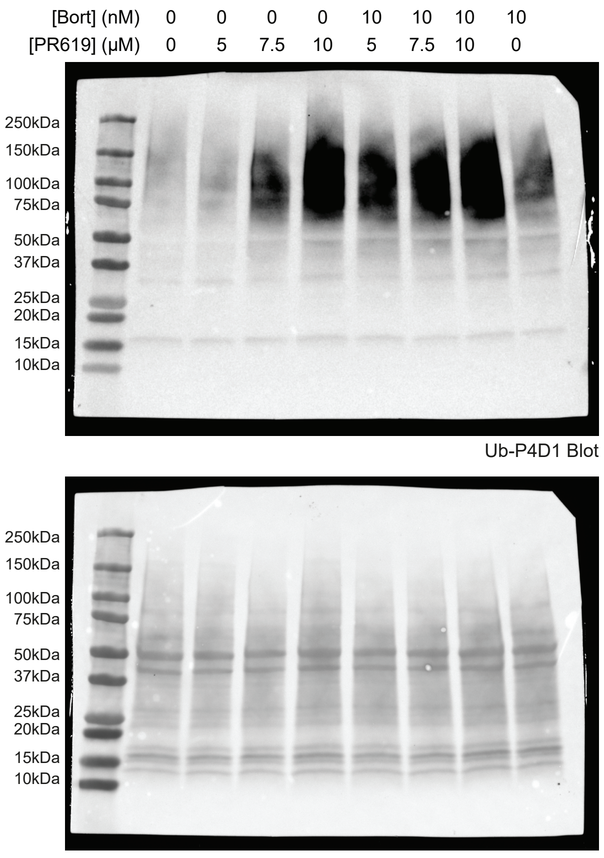

Supplementary Figure 6A: NRF2 Blot

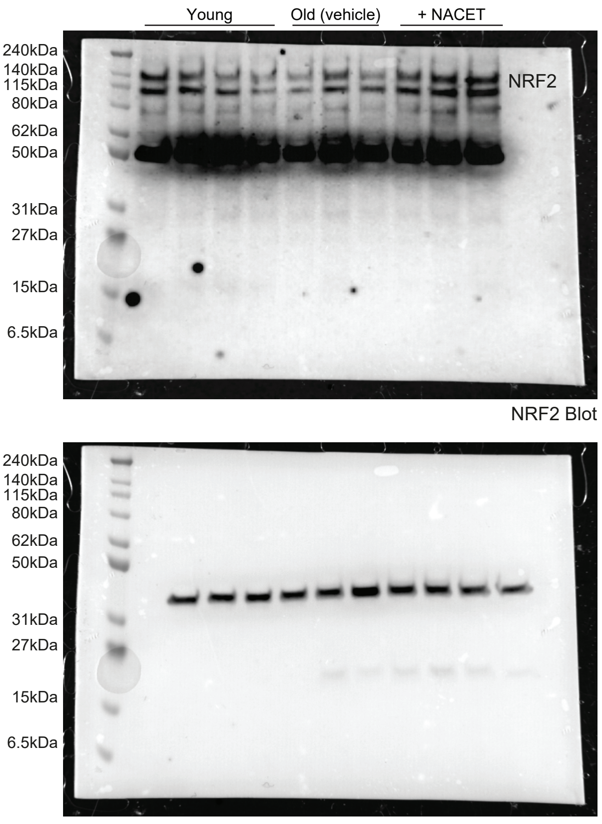

Supplementary Figure 6C: Total and K48 ubiquitylated proteins blot of NACET-treated aged brain lysates

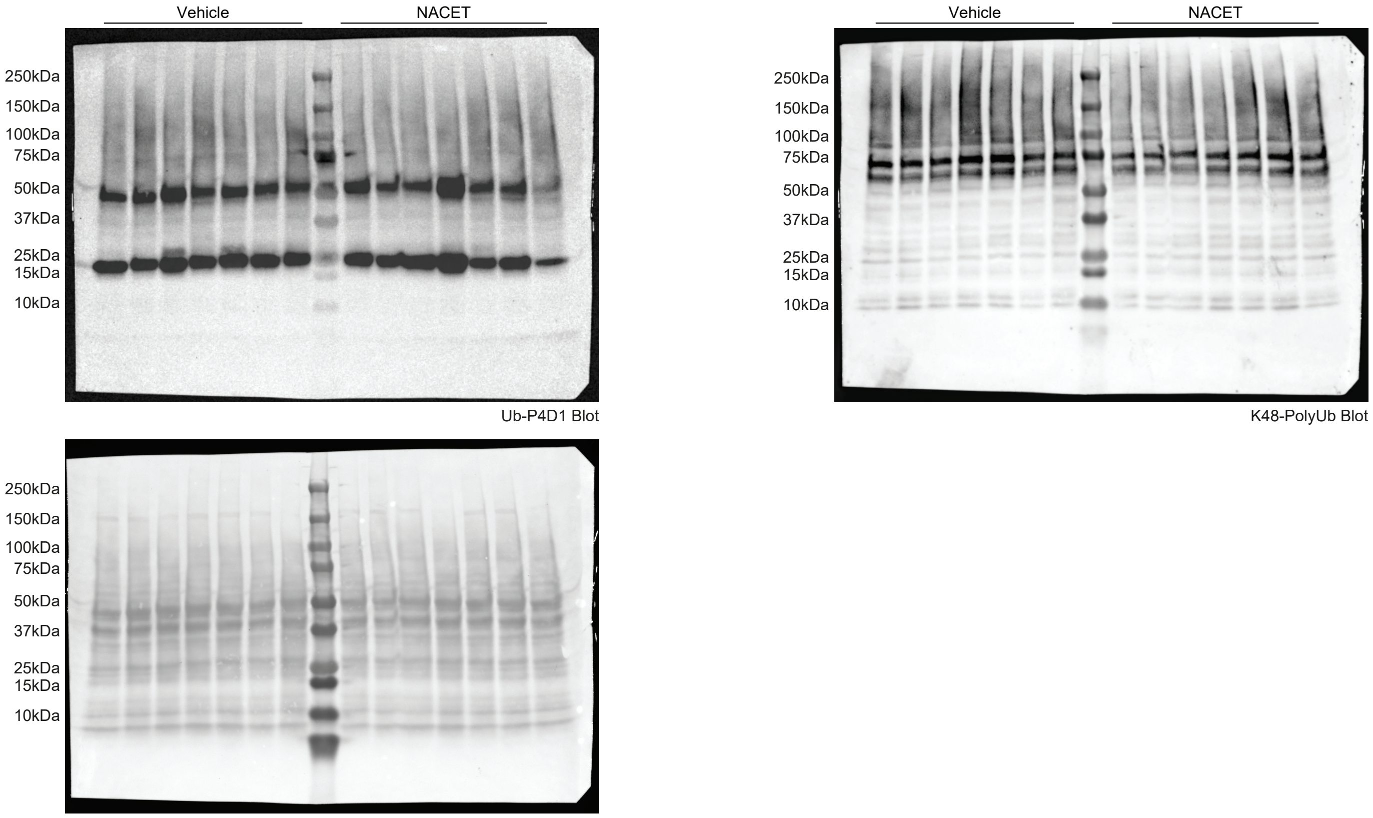

Supplement: Supplementary file 1 — Supplementary Information [file 41467_2026_71921_MOESM1_ESM.pdf]
