# Supplementary material for: Oxidative stress causes a reversible decrease of deubiquitylases activity in old vertebrate brains
Source: Nat Commun. 2026 Apr 21;17:3653. doi: 10.1038/s41467-026-71921-y (PMC13100221; doi:10.1038/s41467-026-71921-y)
Supplement: Supplementary file 2 — Description of Additional Supplementary Files [file 41467_2026_71921_MOESM2_ESM.pdf]

Description of Additional Supplementary Files

File Name: Supplementary Data 1

Description: Chemoproteomics of DUBs identified and their abundance in mice and killifish brains during aging. Related to Figure 1, Figure 2, Figure S1, and Figure S2.

File Name: Supplementary Data 2

Description: Ubiquitylome and proteome changes in human iPSC-derived iNeurons induced by global DUB inhibitor (PR619), USP7-specific inhibitor (P5091), and proteasome inhibitor (Bortezomib (Bort)) treatment. Related to Figure 3, Figure S3, and Figure S4.

File Name: Supplementary Data 3

Description: Absolute quantification of ubiquitin-chain linkages using AQUA-PRM in DUB- and proteasome-inhibited iNeurons and NACET-treated aged mouse brains. Related to Figure 4, Figure 5, and Figure S6.
